# Nanotechnology and Dental Implants

**DOI:** 10.1155/2010/915327

**Published:** 2010-12-28

**Authors:** Sandrine Lavenus, Guy Louarn, Pierre Layrolle

**Affiliations:** ^1^Inserm U957, Bone Resorption Physiopathology and Primary Bone Tumors Therapy, Faculty of Medicine, University of Nantes - 1 rue Gaston Veil, 44035 Nantes cedex 1, France; ^2^Institut des Matériaux Jean Rouxel (IMN), CNRS, Université de Nantes - 2, rue de la Houssinière, 44322 Nantes cedex 3, France; ^3^ERT2004, Faculty of Dental Surgery, University of Nantes - Place Alexis Ricordeau, 44042 Nantes Cedex 01, France

## Abstract

The long-term clinical success of dental implants is related to their early osseointegration. This paper reviews the different steps of the interactions between biological fluids, cells, tissues, and surfaces of implants. Immediately following implantation, implants are in contact with proteins and platelets from blood. The differentiation of mesenchymal stem cells will then condition the peri-implant tissue healing. Direct bone-to-implant contact is desired for a biomechanical anchoring of implants to bone rather than fibrous tissue encapsulation. Surfaces properties such as chemistry and roughness play a determinant role in these biological interactions. Physicochemical features in the nanometer range may ultimately control the adsorption of proteins as well as the adhesion and differentiation of cells. Nanotechnologies are increasingly used for surface modifications of dental implants. Another approach to enhance osseointegration is the application of thin calcium phosphate (CaP) coatings. Bioactive CaP nanocrystals deposited on titanium implants are resorbable and stimulate bone apposition and healing. Future nanometer-controlled surfaces may ultimately direct the nature of peri-implant tissues and improve their clinical success rate.

## 1. Introduction

**Figure 1:**
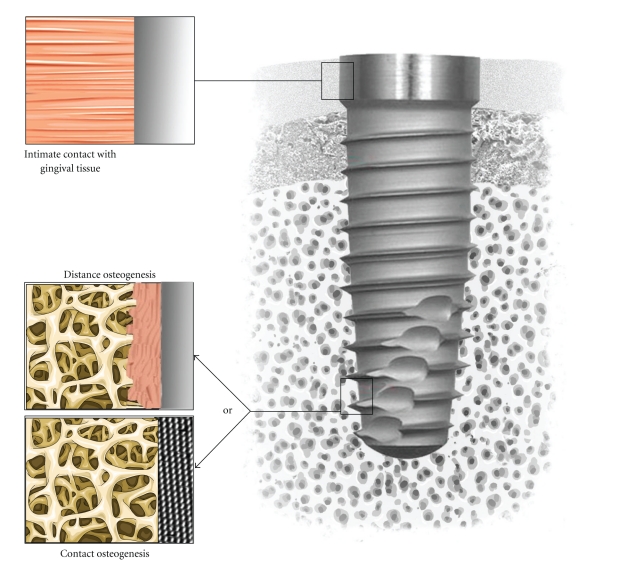
Tissue integration of dental implant. Note the intimate contact with gingival tissue in the upper part and the desired contact osteogenesis in the tapered lower part rather than distance osteogenesis.

Implants are commonly used in dental surgery for restoring teeth. One of the challenges in implantology is to achieve and maintain the osseointegration as well as the epithelial junction of the gingival with implants. An intimate junction of the gingival tissue with the neck of dental implants may prevent bacteria colonisations leading to peri-implantitis while direct bone bonding may ensure a biomechanical anchoring of the artificial dental root (Figure 1). 

The first step of the osseointegration of implants is called primary stability and is related to the mechanical anchorage, design of implants, and bone structure [1]. This primary interlock decreases with time at the benefit of the secondary anchorage, which is characterized by a biological bonding at the interface between bone tissues and implant surface. Between the primary mechanical and secondary biological anchorage, a decrease of implant stability could be observed. Many studies have attempted to enhance the osseointegration of implants by various surface modifications. The aim is to provide metal implants with surface biological properties for the adsorption of proteins, the adhesion and differentiation of cells, and tissue integration. These biological properties are related to chemical composition, wettability, and roughness of metal implants surfaces. However, the control of these surface properties at the protein and cell levels, thus in the nanometre range, remains a challenge for researchers and dental implants manufacturers.

Nanotechnologies may produce surfaces with controlled topography and chemistry that would help understanding biological interactions and developing novel implant surfaces with predictable tissue-integrative properties [2, 3]. Various processing methods derived from the electronic industry such as lithography, ionic implantation, anodization, and radio frequency plasma treatments may be applied to the surfaces of dental implants to produce controlled features at the nanometer scale. These surfaces may then be screened by using high throughput biological assays *in vitro*. For instance, specific protein adsorption, cell adhesion, and differentiation of stem cells should be studied in relation to the surface properties. This approach may define the ideal surface for a specific biological response. Following *in vitro* screening, nanostructured surfaces may then be tested in animal models to validate hypothesis in a complex *in vitro* environment. 

**Figure 2:**
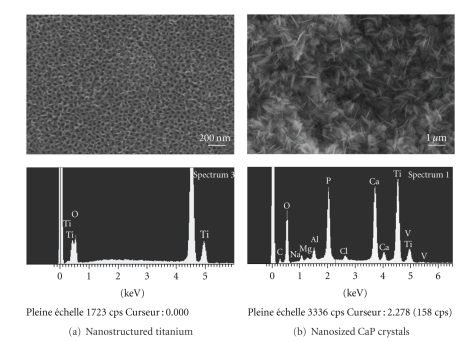
Scanning electron micrographs and energy dispersive analysis for X-ray of (a) nanostructured titanium surface obtained by anodization and (b) nanosized thin calcium phosphate (CaP) coating on titanium produced by electrochemical deposition. Note the regular array of TiO2 nanopores of approximately 100 nm in diameter and the nanosized CaP crystals on titanium surfaces.

New coating technologies have also been developed for applying hydroxyapatite and related calcium phosphates (CaP), the mineral of bone, onto the surface of implants (Figure 2). Many studies have demonstrated that these CaP coatings provided titanium implants with an osteoconductive surface [4, 5]. Following implantation, the dissolution of CaP coatings in the peri-implant region increased ionic strength and saturation of blood leading to the precipitation of biological apatite nanocrystals onto the surface of implants. This biological apatite layer incorporates proteins and promotes the adhesion of osteoprogenitor cells that would produce the extracellular matrix of bone tissue. Furthermore, it has been also shown that osteoclasts, the bone resorbing cells, are able to degrade the CaP coatings through enzymatic ways and created resorption pits on the coated surface [5]. Finally, the presence of CaP coatings on metals promotes an early osseointegration of implants with a direct bone bonding as compared to noncoated surfaces. The challenge is to produce CaP coatings that would dissolve at a similar rate than bone apposition in order to get a direct bone contact on implant surfaces.

This paper reviews the different steps of the interactions between biological fluids, cells, tissues, and surfaces of implants. Recent nanoscale surface modifications and calcium phosphate coating technologies of dental implants are discussed. The sequence of biological events in relation to surface properties is related. Mechanisms of interaction with blood, platelets, hematopoietic, and mesenchymal stem cells on the surface of implants are described. These early events have shown to condition the adhesion, proliferation, and differentiation of cells as well as the osseointegration of implants. Future implant surfaces may improve the tissue-integrative properties and long-term clinical success for the benefits of patients.

## 2. Nanoscale Surface Modifications

Surfaces properties play a determinant role in biological interactions. In particular, the nanometer-sized roughness and the chemistry have a key role in the interactions of surfaces with proteins and cells. These early interactions will in turn condition the late tissue integration. In this prospect, different methods have been reported for enhancing bone healing around metal implant [2, 6].

Modifying surface roughness has been shown to enhance the bone-to-implant contact and improve their clinical performance [2, 7]. Grit blasting, anodisation, acid etching, chemical grafting, and ionic implantation were the most commonly used methods for modifying surface roughness of metal implants. Combinations of these techniques could be used such as acid etching after grit-blasting in order to eliminate the contamination by blasting residues on implant surfaces. This grit blasting residue may interfere with the osteointegration of the titanium dental implants [8–10]. It has been shown that grit-blasting with biphasic calcium phosphate (BCP) ceramic particles gave a high average surface roughness and particle-free surfaces after acid etching of titanium implants. Studies conducted both *in vitro* and *in vivo* have shown that BCP grit-blasted surfaces promoted an early osteoblast differentiation and bone apposition as compared to mirror-polished or alumina grit-blasted titanium [11, 12]. Anodization is a method commonly used to obtain nanoscale oxides on metals including titanium [13, 14]. By adjusting the anodization condition such as voltage, time, and shaking, nanoscale properties could be controlled. Shankar et al. [15] have reported that the diameters of the nanotubes could be modified to a range from 20 to 150 nm in modifying voltage conditions. On the other hand, Kang et al. [16] found that TiO_2_ nanotube arrays were more uniform on electro-polished than machined titanium. Moreover, TiO_2_ nanotubes on Ti improved the production of alkaline phosphatase (ALP) activity by osteoblastic cells. In particular, nanotubes with a diameter of 100 nm upregulated level of ALP activity as compared to 30–70 nm diameter nanotube surfaces [17]. Since ALP is a marker of osteogenic differentiation, these surfaces may demonstrate enhanced bone tissue integrative properties.

Another approach for improving osseointegration of dental implants is to apply a CaP coating having osteoconductive properties [18–20]. Different methods have been developed to coat metal implants with CaP layers such as plasma spraying, biomimetic and electrophoretic deposition. Nevertheless, plasma-sprayed HA-coated dental implants have been related to clinical failures due to coating delimitation and heterogeneous dissolution rate of deposited phases. An electrochemical process which consists of depositing calcium phosphate crystals from supersaturated solutions has been proposed for coating titanium implants with calcium phosphate layers [21, 22]. Upon implantation, these CaP coatings dissolve and release Ca^2+^ and HPO_4_^2−^) increasing saturation of blood in the peri implant region. This dissolution led to the precipitation of biological apatite nanocrystals with the incorporation of various proteins. This biological apatite layer will promote cell adhesion, differentiation into osteoblast, and the synthesis of mineralized collagen, the extracellular matrix of bone tissue. In addition to dissolution, osteoclast cells are also able to resorb the CaP coatings and activate osteoblast cells to produce bone tissue. As a result, these CaP coatings promote a direct bone-implant contact without an intervening connective tissue layer leading to a proper biomechanical fixation of dental implants.

## 3. Interactions of Surface Dental Implants with Blood

**Figure 3:**
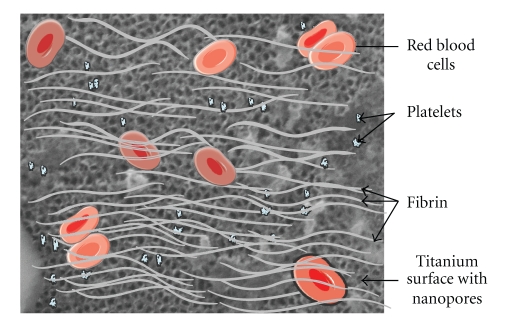
Interactions of surface of dental implants with blood. Note the numerous proteins, red blood cells, and activated platelets that lead to blood clotting on implants.

During surgery, blood vessels are injured and, thus, dental implant surfaces interact with blood components (Figure 3). Various plasma proteins get adsorbed on the material surface within a minute. Platelets from blood interact also with the implant surface. Plasma proteins modified the surface while activated platelets are responsible of thrombus formation and blood clotting. Subsequently, the migrations of various cell types interact with the surface through membrane integrin receptors. These early events occur prior to peri implant tissue healing.

**Figure 4:**
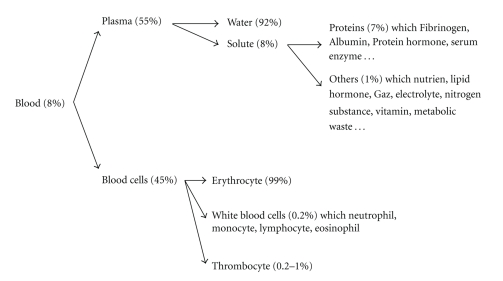
Scheme showing blood composition and components that primary interact with surface of dental implants.

Plasma contains dissolved substances such as glucose, amino acids, cholesterols, hormones, urea, and various ions (Figure 4). Most of these components are needed for the viability of cells and tissues. All of these blood substances could interact with implant surface thus modifying their chemical properties like charge or hydrophobicity.

Blood interactions with implants lead to protein adsorption, which is dependent on the surface properties of the material and occurs through a complex series of adsorption and displacement steps known as the Vroman effect [23]. A hydrophilic surface is better for blood coagulation than a hydrophobic surface. Consequently, dental implants manufacturers have developed high hydrophilic and rough implant surfaces which in turn exhibited better osteointegration than conventional ones [24]. Adsorption of proteins such as fibronectin, vitronectin on surface of dental implants could promote cell adhesion by cell-binding RGD domain (arg-gly-asp). This RGD sequence interacts with integrin present on the cell membrane [25]. Interactions between cell membrane integrins and proteins coated onto implant surface play a key role in adhesion of many cells types. After proteins absorption, the osteointegration is characterized by platelets adhesion and fibrin clots formation at the injured blood vessels site. It has been shown that implants in contact with platelet-rich plasma (PRP) with a platelet concentration of approximately 1,000,000 protein/*μ*L have a positive effect on osteointegration. At lower concentrations of PRP, the effect was not optimal, while higher concentrations resulted in a paradoxically inhibitory effect of bone regeneration. Other studies were not in agreement with this PRP beneficial effect on the osseointegration of dental implants [26]. The assessment of bioactivity of surface-treated dental implants should be tested *in vitro* using biological fluids containing blood components [2].

## 4. Interactions between Surfaces and Mesenchymal Stem Cells

Following blood clotting around dental implants, several cells interact with surfaces for tissue healing. Mesenchymal stem cells (MSCs) attracted to the injured site by chemotactic factors have a determinant role in peri implant tissue healing.

### 4.1. Origin of Mesenchymal Stem Cells

Mesenchymal stem cells (MSCs) are stem cells derived from somatic tissue which can be differentiated into mesenchymal lineages such as bone, cartilage, fat, and skin. In addition, MSCs are present in many conjunctive tissues and blood at low concentrations serving as a sort of internal repair system. Mesenchymal stem cells are distinguished from other cell types by two important characteristics. First, they are unspecialized cells able to renew themselves through cell division, sometimes after long periods of inactivity. Second, under certain physiologic or experimental conditions, they can be induced to become tissue- or organ-specific cells with special functions. MSCs have high proliferative and multipotent capacity leading to differentiated cells under the guidance of various cues or niches. MSCs are conventionally defined as adherent, nonhematopoietic cells expressing markers such as CD13, CD29, CD44, CD54, CD73, CD90, CD105, and CD166, and being negative for CD14, CD34, and CD45 [27, 28]. While originally identified in the bone marrow [29], MSCs have been extracted from numerous tissues including adipose [30, 31], heart [32], dental pulp [33], peripheral blood [34], and cord blood [35]. One of the major properties of MSCs is their ability to differentiate into various cells like adipocytes [36], chondrocytes [30], osteoblasts [37], neurons [38, 39], muscles [39, 40], and hepatocytes [41] *in vitro* after treatment with induction agents.

### 4.2. Migration, Adhesion, and Proliferation

**Figure 5:**
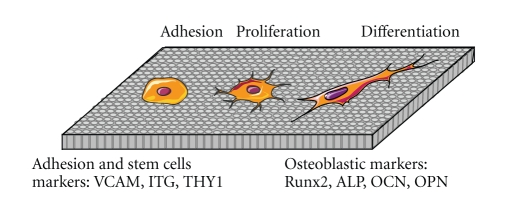
Scheme of the adhesion, proliferation, and differentiation of mesenchymal stem cells on nanostructured surfaces. The adhesion of stem cells is characterized by the expression of cell surface markers (VCAM, ITG, and THY1) while phenotypic markers (Runx2, ALP, OCN, and OPN) are specific to their osteoblastic differentiation (OCN: osteocalcin; OPN: osteopontin).

The integration of implant with neighboring bone and gingival tissue depends on successful crosstalk between old tissue and implant surface. The challenge in dental implant research is the capability of the surface to guide cells colonization and differentiation. Cell migration, adhesion, and proliferation on implant surfaces are a prerequisite to initiate the tissue regeneration (Figure 5). Authors have shown that some factors present in tissues and secreted during the inflammatory phase are able to attract MSCs to the injured site [42, 43]. MSCs migration and proliferation were stimulated *in vitro* by many growth factors including PDGF [44, 45], EGF [45, 46], VEGF [47], TGF-*β* [44, 48], and BMP-2 and BMP-4 [44, 47]. These factors are certainly released in the injured sites by cells involved in tissue healing. Furthermore, plasma clot serves as storage to fibrin molecules and releases system for a variety of bioactive factors including growth factors that attract and differentiate MSCs into specific lineages [49–51]. The platelet factors are well known to stimulate the proliferation of MSCs [52]. The formation of a clot matrix with a potent chemoattractive factor like PDGF, EGF, or fibrin may further enhance MSCs numbers and peri implant tissue healing surface. Moreover, the plasma clot in contact with implant surface represents a three-dimensional microporous structure that allows diffusion of regulatory factors [53, 54] and is involved in the migration, proliferation, and differentiation of MSCs. After MSC recruitment in the injured site, cells adhere on the local extracellular matrix as well as on the implant surface beginning an extensive proliferation in order to build up new tissue. Again, surface modifications of implants in the nanometer range condition the biological responses.

### 4.3. Differentiation

In the microenvironment, MSCs are stimulated by some specific factors to differentiate into the adequate cell line. Under the influence of these factors, MSCs switch to osteoblastic cells in contact to bone tissue while they differentiate into fibroblastic lineage in the gingival tissue region. These two differentiation pathways are in concurrence around dental implants. In some cases, implants are encapsulated by fibrous tissue due to the proliferation and differentiation of MSCs into fibroblastic cells. In response to cytokine, fibroblasts migrate and generate a capsule of collagen, the first step in generation of gingival tissue or rejection on contact to bone. This fibrous capsule prevents bonding between implant surface and juxtaposed bone and causes a failure of the implant [55]. On the other hand, both the differentiation of MSCs into fibroblastic lineage and the fibroblastic adhesion are desired in the gingival upper part of dental implants. Fibroblasts adhesion has been shown to be lower on nanoscale surface compared to conventional surfaces [56]. Moreover, nanometer size features have been shown to decrease fibroblast adhesion and proliferation [57, 58]. The micro- and nanoscale surface properties of metal implant, including chemistry, roughness, and wettability, could affect bone formation [59]. Numerous treatments such as machining, grit-blasting, Ti/HA plasma spray, chemical etching, and anodization are available to modify the implant surface. Research has specifically demonstrated that nanorough Ti [60] and nanostructured Ti can enhance osteoblast adhesion and differentiation compared to their nanosmooth control [61]. Furthermore, surfaces with micro- and nanopores have shown to enhance greatly osseointegration [62, 63]. Surface properties may control the steps of adhesion, proliferation, and differentiation of MSCs and, thus, condition tissue integration.

## 5. Tissue Integration

Brånemark et al. [64] described the osseointegration as a direct structural and functional bone to implant contact under load. As previously discussed, the biological events occurring at the tissue-implant interface are influenced by the chemistry, topography, and wettability of dental implant surfaces. The challenge in developing new implant surface consists in increasing the clinical success rate as well as decreasing the tissue healing time for immediate loading of implants, particularly in aesthetic situations [65–67]. One of the objectives is to develop implant surface having predictable, controlled, and guided tissue healing. For instance, surfaces that promote contact osteogenesis rather than distance osteogenesis would be desired in bony site while intimate fibrous tissue healing in gingival tissue (Figure 1). In order to enhance this intimate contact between tissues and implant, surface treatments at the nanometer scale have been performed on metal implants and tested in various animal models. Implant surface with various roughnesses have been used to increase the total area available for osteo-apposition. Kubo et al. [65] observed a substantial increase by 3.1 times in bone-titanium interfacial strength by Ti nanotube (300 nm) at 2 weeks of implantation in femur rats. These results suggest the establishment of nanostructured surfaces for improved osteoconductivity. Moreover, Ogawa et al. [68] have prepared Ti nanostructure by physical vapour deposition and tested their osseointegration in femur of rats. They found an increased surface area by up to 40 % and a greater strength of osseointegration for the nanostructured compared to an acid-etched surface. Some authors have correlated the initial events in bone formation adjacent to surface with the long-term tissue response to these materials in humans [69, 70]. 

**Figure 6:**
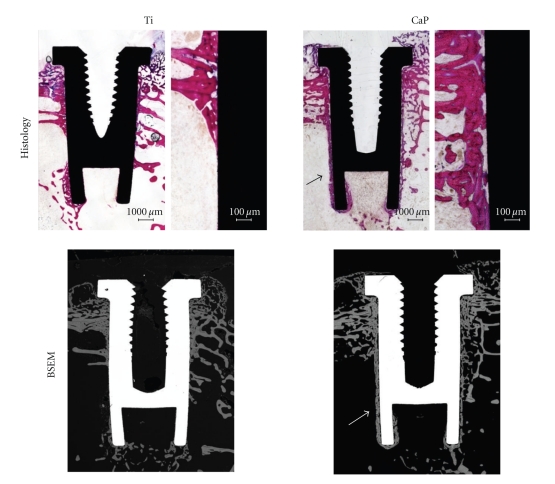
Micrographs showing the osteointegration of bare titanium- (Ti-) and calcium phosphate- (CaP-) coated implants after implantation in femoral condyles of rabbits for 4 weeks. Note the direct bone apposition on CaP-coated implants (arrows) on both histology (basic fuchsin, toluidine blue staining) and back-scattered electron microscopy (BSEM) images.

By mimicking the chemical composition of natural bone, hydroxyapatite and CaP coatings on Ti greatly enhance osteointegration. As shown in Figure 6, a greater direct bone apposition was observed on CaP-coated than on bare Ti-coated implants. During the bone healing process, calcium and phosphate ions are released from the CaP coating in the peri-implant region and saturate body fluids to precipitate a biological apatite, which serves as a substrate for osteoblastic cells producing bone tissue. Several authors have shown the benefit of using CaP-coated titanium implants for improving the osteointegration [71, 72]. In particular, Le Guehennec et al. [20] have studied the osteointegration of four implant surfaces in the femoral epiphyses of rabbits after 2 and 8 weeks of healing. In this study, the bone-implant contact and bone growth inside the chambers were compared for four different implant surfaces and shown that biomimetic coating method may enhance the bone apposition onto titanium. In order to prevent coating delamination and implant loosening, the CaP coating should dissolve or degrade under osteoclastic activity at a similar rate than bone apposition. The final result should be a direct bone-implant coating without the presence of fibrous tissue. Another advantage of these CaP coatings is related to their preparation by biomimetic methods at physiological temperature and pH from simulated body fluids. CaP crystals have characteristics that resemble bone mineral in terms of size and composition. Furthermore, it is possible to incorporate biologically active drugs such as antibiotics or growth factors during the precipitation of CaP coatings on Ti implants [73]. These molecules could be locally and gradually released in the peri implant reguion for either preventing bacterial infections or stimulating bone growth.

## 6. Conclusion

Many reports have shown that nanometer-controlled surfaces have a great effect on early events such as the adsorption of proteins, blood clot formation, and cell behaviours occurring upon implantation of dental implants. These early events have an effective impact on the migration, adhesion, and differentiation of MSCs. Nanostructured surfaces may control the differentiation pathways into specific lineages and ultimately direct the nature of peri-implant tissues. Despite an active research in dental implants, the ideal surface for predictive tissue integration remains a challenge.
